# Sherlock-Genome: an R Shiny application for genomic analysis and visualization

**DOI:** 10.1186/s12864-024-11147-8

**Published:** 2025-01-16

**Authors:** Alyssa Klein, Jun Zhong, Maria Teresa Landi, Tongwu Zhang

**Affiliations:** Division of Cancer Epidemiology and Genetics, National Cancer Institute, Bethesda, MD USA

**Keywords:** Cancer Genomics, Whole Genome Sequencing, R Shiny, Data Visualization, Bioinformatic Pipeline

## Abstract

**Motivation:**

Next-generation sequencing technologies, such as whole genome sequencing (WGS), have become prominent in cancer genomics. However, managing, visualizing, and integratively analyzing WGS results across various bioinformatic pipelines remains challenging, particularly for non-bioinformaticians, hindering the usability of WGS data for biological discovery.

**Results:**

We developed Sherlock-Genome, an R Shiny app for data harmonization, visualization, and integrative analysis of WGS-based cancer genomics studies. Following FAIR principles, Sherlock-Genome provides a platform and guidelines for managing and sharing finalized sample-level WGS analysis results, enabling users to upload results, inspect analyses locally, and perform integrative analyses. It includes modules for major cancer genomic analyses, allowing interactive data visualizations and integrative analyses with other data types. Sherlock-Genome supports both local and cloud deployment, facilitating the sharing of results for related publications. This tool has the potential to be widely adopted in cancer genomics, significantly enhancing the accessibility and usability of sample-level WGS analysis results for comprehensive biological discovery and research advancements.

**Availability and implementation:**

The source code and installation instructions for Sherlock-Genome can be accessed via Github https://github.com/xtmgah/Sherlock-Genome. Documentation and data requirements for user project data can also be found on the same GitHub page.

**Supplementary information:**

The online version contains supplementary material available at 10.1186/s12864-024-11147-8.

## Introduction

The rapid advancements in next-generation sequencing (NGS) technologies, particularly whole genome sequencing (WGS), have revolutionized the field of cancer genomics [1, 2]. These technologies have become increasingly accessible due to their reduced costs and enhanced efficiency, making them integral to contemporary genomics studies. However, the sheer volume and complexity of WGS data present significant challenges in data analysis, management, and visualization, especially for researchers without a bioinformatics background [1, 3, 4]. This gap often impedes the full utilization of WGS data for biological discoveries.

In cancer genomics, various analyses can be performed using WGS data. Current solutions for handling sample-level analytical results from WGS data are often limited in scope, focusing on singular types of analyses, and lacking an efficient platform for data management and downstream analysis. These tools frequently fail to provide comprehensive frameworks for integrative analyses that incorporate various WGS-based bioinformatic pipelines. For example, the widely used platform cBioPortal focuses on whole exome sequencing [5] and lacks the capability to handle analytical results from WGS data. This underscores the critical need for a robust, user-friendly platform that adheres to the FAIR (Findability, Accessibility, Interoperability, and Reusability) principles, enabling efficient and effective utilization of WGS data.

In response to this need, we developed Sherlock-Genome, an R Shiny application designed to facilitate the harmonization, visualization, and integrative analysis of WGS data in cancer genomics. Sherlock-Genome offers a versatile platform that supports both local and cloud-based deployment, providing detailed guidelines for data preparation and management. Users can upload their WGS results, perform integrative analyses, and visualize data interactively through an intuitive interface. The application includes modules for various cancer genomic analyses, allowing researchers to conduct comprehensive studies that integrate clinical and epidemiological data.

## Implementation

**Fig. 1:**
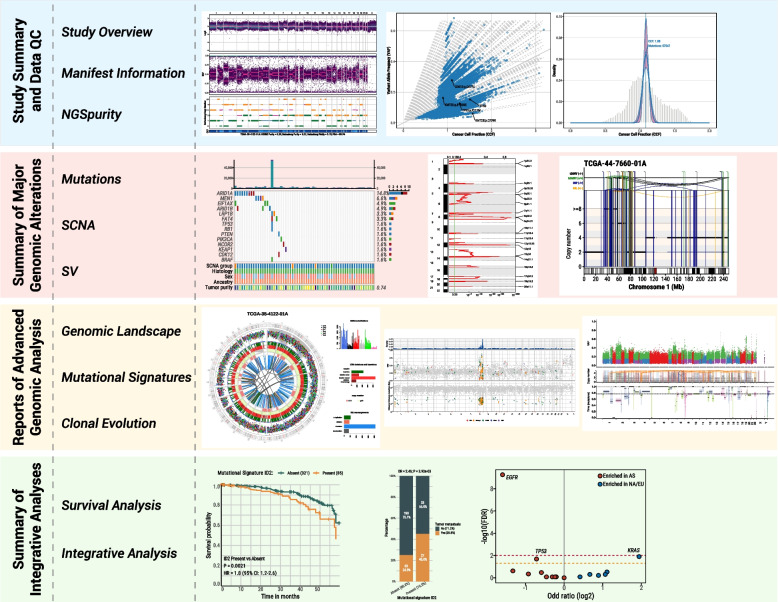
Overview of Major Modules and Example Visualizations Supported by Sherlock-Genome. Sherlock-Genome includes 11 modules for comprehensive genomic analysis, grouped into study summary and data quality control (QC), major genomic alterations, advanced genomic analysis, and integrative analyses. Representative visualizations for each module are shown. I) *Study Summary and Data Quality Control (QC)*: Visualizations generated by NGSpurity include somatic copy number alteration (SCNA) plots (logR/BAF/copy number status/clonality), relationships between cancer cell fraction (CCF) and variant allele frequency (VAF), and distributions of CCF and clone clustering using a Dirichlet process. II) *Summary of Major Genomic Alterations*: Visualizations include an oncoplot of top mutated genes and genomic features, recurrent focal amplifications identified by the GISTIC2 algorithm, and rearrangement and copy number visualizations using ReConPlot. III) *Reports of Advanced Genomic Analysis*: Visualizations include circos plot representations of major genomic alterations (SNVs, indels, CNVs, and rearrangements), rainfall plot illustrating inter-mutational distance for clustered somatic mutations, and timing of somatic mutations relative to clonal and subclonal copy number states using MutationTimeR. IV) *Summary of Integrative Analyses*: Visualizations include survival analyses based on the status of one selected genomic alteration or feature, enrichment analysis between two genomic alterations or features using Fisher's exact test, and logistic regression-based associations between genomic alterations and one selected genomic feature. Additionally, other sample-level cancer genomic analysis results (*e.g.,* mutational signature presence or activity) can be incorporated for integrative analyses. Detailed descriptions of these functions and additional features are available in the respective modules of the Sherlock-Genome Shiny app

Sherlock-Genome was designed to facilitate the investigation of WGS analytical results by researchers without requiring strong programming skills. To this end, we developed Sherlock-Genome, a Shiny app built with R Shiny version 1.8.0, enabling genomic analysis and visualization of WGS data at the sample level. It contains several modules with a range of functions that are easy to use via a user-friendly interface, making it accessible even to those without an advanced background in bioinformatics (Fig. 1). All plots in the Sherlock-Genome app can be downloaded as publication-ready figures from each module.

This application recommends pipelines for WGS analyses and provides detailed guidelines on data preparation. Users can upload sample-level results from various WGS pipelines into Sherlock-Genome, where the data are harmonized and integrated. Data harmonization involves standardizing results from different pipelines and consolidating genomic alterations or features from multiple sources at the sample level. The app uses the sample ID (“Tumor_Barcode”) as the key identifier to join and manage these data, enabling seamless inspection, visualization, and integrative analysis. Independent modules in the app allow users to generate a variety of interactive data inspection and visualization images and perform numerous integrative analyses using clinical and epidemiological data.

An example of Sherlock-Genome with a subset of The Cancer Genome Atlas (TCGA) Lung Adenocarcinoma (LUAD) samples has been included to demonstrate its capabilities. This dataset includes 10 samples that users can explore when selecting the option to load a provided project in the app. Additionally, the application includes a folder containing data requirements for users to reference when they wish to upload their data. This folder is accessible through the Documentation module and the Data Requirement Info tab, allowing users to explore the different files needed for each module and the specific headers or formatting required for each file. Detailed documentation for the application and a spreadsheet with specific descriptions of data requirements are also available to help users prepare their data correctly.

## Modules for cancer genomic analysis

The independent modules included in Sherlock-Genome are designed to facilitate comprehensive genomic analyses and are grouped into four major categories (Supplementary Table 1). The first category, *Study Summary and Data QC*, provides essential study manifest information, comprehensive data quality control (QC) checks, and summaries of the uploaded WGS data. The WGS QC is generated by our NGSpurity pipeline, which was developed to estimate tumor purity, ploidy, and clonal architecture by integrating somatic copy number alterations (SCNA), single nucleotide variants (SNV), and cancer cell fractions (CCF) [6]. The second category, *Summary of Major Genomic Alterations*, allows users to explore and visualize SNV, SCNA, and structural variants (SV). Sherlock-Genome supports various bioinformatic pipelines to analyze these genomic alterations and incorporates visualization functions from original tools such as MAFtools for oncoplots, tumor mutational burden (TMB) comparisons with TCGA, and lollipop plots [7]. For SCNA, the application also supports clustering arm-level SCNA and visualizing recurrent focal-level SCNA using the GISTIC2 algorithm [8]. The ReconPlot [9] is included to visualize complex genomic rearrangements. The third category, *Reports of Advanced Genomic Analyses*, enables the exploration of advanced genomic features, including clustered mutations generated by SigProfilerCluster [10] and complex genomic landscapes visualized by the Signature Tools Lib Package [11]. Major mutational signature data (e.g., signature presence or signature activity), regardless of the computational method used, can be incorporated into Sherlock-Genome as a genomic feature for integrative analyses. Additionally, mutationTimeR [12] is used to time somatic mutations relative to clonal and subclonal copy number states. The fourth category, *Summary of Integrative Analyses*, encompasses functions for integrative analyses, such as survival analysis, enrichment analysis using Fisher's exact test, and association testing (univariate and multivariable). Specific data visualizations for these integrative analyses include bar plots, box plots, volcano plots, and genomic word cloud plots. Compared to the WES-based data portal cBioPortal, Sherlock-Genome offers unique advantages in data visualization and analysis tailored for WGS datasets, including supporting complex genomic events and advanced genomic features.

## Conclusions

Sherlock-Genome's ability to streamline complex genomic workflows and its potential for broad adoption make it a valuable tool for enhancing the accessibility and usability of WGS data. By bridging the gap between sophisticated bioinformatic analyses and user-friendly interfaces, Sherlock-Genome stands to significantly advance research in cancer genomics and foster new biological discoveries.

However, as an R Shiny application, Sherlock-Genome may encounter performance limitations when handling very large datasets. While the tool is optimized for sample-level analysis and visualization, its performance may be affected by the size and complexity of the data. Users are encouraged to keep these limitations in mind when working with particularly large datasets.

## Availability and requirements

Project name: Sherlock-Genome.

Project home page: https://github.com/xtmgah/Sherlock-Genome.

Operating system(s): Platform independent.

Programming language: R.

Other requirements: Dependent on R packages.

License: MIT.

Any restrictions to use by non-academics: None.

## Supplementary information

Supplementary Material 1 Supplementary Table 1. Module breakdown within *Sherlock-Genome* and comparison to cBioPortal. Module categories and the specific modules that correspond to a respective category, along with a brief description of the module’s purpose.

## Data Availability

No datasets were generated or analysed during the current study.
